# Dynamics of social corrections to peers sharing COVID-19 misinformation on WhatsApp in Brazil

**DOI:** 10.1093/jamia/ocab219

**Published:** 2021-11-22

**Authors:** Santosh Vijaykumar, Daniel T Rogerson, Yan Jin, Mariella Silva de Oliveira Costa

**Affiliations:** 1 Department of Psychology, Northumbria University, Newcastle upon Tyne, UK; 2 COCO Lab, Northumbria University, Newcastle upon Tyne, UK; 3 Department of Advertising and Public Relations, University of Georgia, Athens, USA; 4 Communication Office, Oswaldo Cruz Foundation, Brasilia, Brazil

**Keywords:** misinformation, COVID-19, social media, correction, behavior, Brazil

## Abstract

**Objective:**

Online COVID-19 misinformation is a serious concern in Brazil, home to the second-largest WhatsApp user base and the second-highest number of COVID-19 deaths. We examined the extent to which WhatsApp users might be willing to correct their peers who might share COVID-19 misinformation.

**Materials and Methods:**

We conducted a cross-sectional online survey using Qualtrics among 726 Brazilian adults to identify the types of social correction behaviors (SCBs) and health and technological factors that shape the performance of these behaviors.

**Results:**

Brazil’s WhatsApp users expressed medium to high levels of willingness to engage in SCBs. We discovered 3 modes of SCBs: correction to the group, correction to the sender only, and passive or no correction. WhatsApp users with lower levels of educational attainment and from younger age groups were less inclined to provide corrections. Lastly, the perceived severity of COVID-19 and the ability to critically evaluate a message were positively associated with providing corrections to either the group or the sender.

**Discussion:**

The demographic analyses point to the need to strengthen information literacy among population groups that are younger with lower levels of educational attainment. These efforts could facilitate individual-level contributions to the global fight against misinformation by the World Health Organization in collaboration with member states, social media companies, and civil society.

**Conclusion:**

Our study suggests that Brazil’s WhatsApp users might be willing to actively respond with feedback when exposed to COVID-19 misinformation by their peers on small-world networks like WhatsApp groups.

## INTRODUCTION 

Online misinformation, defined as “any health-related claim of fact that is false based on current scientific consensus,”^1^ has posed barriers to the promotion of preventive behaviors and caused social unrest during the COVID-19 pandemic. This global problem has invited a range of responses from several stakeholders such as social media companies and the World Health Organization to developers of misinformation games and news, media, and information literacy initiatives.^2–5^ However, what is less understood is the role that the vast population of 4.48 billion social media users^6^ could themselves play in tackling the issue of misinformation. Our paper focuses on the role and extent of WhatsApp users’ willingness to correct their social peers who might knowingly or unwittingly share COVID-19 misinformation on this popular messaging platform. We first present the conceptual ideas that informed our study, describe the study context, detail the methods, present our findings, and discuss their implications for theory, policy, and practice surrounding the management of online health misinformation that has now returned to thwart COVID-19 vaccination programs.

### Conceptual framework

Online misinformation is corrected through a range of interventions including myth-busting campaigns by public health agencies, algorithm-based news dissemination and by expert organizations, journalists, and fact-checkers.^7–9^ Besides being as effective as algorithmic corrections in correcting misperceptions,^7^ the limited evidence surrounding corrections made by social media users reveals the psychological and technological dynamics at play in influencing this behavior.

WhatsApp users can either *witness* someone else being corrected, *experience* being corrected, or *perform* a correction themselves.^10^ This study focuses solely on the *performance* of correction which we refer to as social correction behaviors (or SCBs). We conceptualize SCB as the voluntary act of feeding back with a deliberate intent to counter perceived misinformation sent by a social media user within one’s WhatsApp network. Given the nascence of this research area, we draw upon contemporary evidence and classic theories of health behavior to identify social psychological factors that inform our conceptual framework underpinning this study.

Research has shown that social media users ignore fake news posts and offer corrections *only* to those with whom they have strong relationships.^11^ This suggests that the closeness of social ties in small networks like WhatsApp groups that are oftentimes comprised of family members or friends might provide a trusted environment, leading members to engage in SCBs. However, verifying and correcting every misleading claim can be a time-consuming process.^12^ While researchers have examined the relationship between time spent on social media and psychological health and well-being,^13^ the extent to which it can contribute to positive behaviors like verifying and correcting information with peers is little understood. Another possible consideration affecting users’ decisions around whether or not to provide corrective feedback to peers could be influenced by how social norms are perceived in small networks like WhatsApp groups. Specifically, social peers have been found to be more accepting of the expression of positive rather than negative emotions across Facebook, Instagram, WhatsApp, and Twitter.^14^ However, when these patterns were disambiguated by platform, expressing negative emotions was found to be most acceptable on WhatsApp.^14^ Placing this evidence in context, it is likely that SCBs in a WhatsApp group might generate negative or positive emotions in the original sender and other members of the group could affect the decision of the user to perform such corrective behaviors.

In addition to the abovementioned technological factors related to exposure to misinformation—critical evaluation of the message, time spent on social media, and the social norms of WhatsApp groups—we suggest that beliefs related to health risks might also affect SCB against misinformation. This argument is supported by extant evidence which shows that the perceived severity of, and perceived susceptibility to COVID-19 contribute to cyberchondria (compulsively seeking online information related to illness or symptoms) with males more likely to share news without verifying its accuracy.^15^ Conversely, we are interested to find out if perceived severity of and susceptibility to COVID-19 may persuade WhatsApp users to issue social corrections to those spreading misinformation in their online groups. Lastly, while it is known that demographic factors like age, sex, income, and education play a role in shaping misinformation beliefs,^8^^,^^16^^,^^17^ the extent to which they affect SCB is unknown.

The decision to perform SCB is thought to be based on a variety of factors such as cognitive and time costs of evaluating the veracity of information, normative perceptions about the appropriateness of such behavior, and variations in our understanding of what constitutes misinformation. These factors were reflected in decisions made by users including whether or not to make a social correction, when to make a social correction (now or later), and to whom to make a social correction (only to the sender or the group). Based on this framework, our study seeks to investigate the following questions.

RQ1: How do WhatsApp users in Brazil engage in social correction behaviors?

RQ2: How do these social correction behaviors differ by demographic factors?

RQ3: How do factors related to misinformation consumption (exposure and beliefs), technology, and health beliefs jointly predict these social correction behaviors?

### Study context

We examine these questions in Brazil, a country with the second-highest number of COVID-19 deaths globally.^18^ Brazil also constitutes the second-largest market with 146.6 million users.^19^ The application allows members to create and subscribe to groups, which function as hives of information sharing among users.^20^ However, its popularity has meant that WhatsApp has been prolifically misused not only for political propaganda during the 2018 national general elections but also as a channel for COVID-19 misinformation.^21^^,^^22^ More than 70% of Brazilians believed in COVID-19 misinformation with WhatsApp being the main vector of misinformation in the country.^23^ Consistent with incidents seen in other countries, misinformation circulating on social media sought legitimacy by false attribution to the Oswaldo Cruz Foundation (Fiocruz), one of the country’s main public health institutions.^24^ Subsequently, Fiocruz disseminated corrective information via online social networks to debunk false information attributed to itself and shared illustrated step-by-step guidelines to help people verify health information on online social networks. Other initiatives to combat COVID-19 misinformation by the Brazilian Health ministry included the Saúde sem Fake News website—not updated since July 2020—where a dedicated team of journalists would factcheck WhatsApp messages sent by users.^25^ They found that messages categorized as false were usually about health authorities, prevention, prognosis of the disease, therapy, and vaccination and that COVID-19 misinformation was placing public behavior and the credibility of Brazil’s National Health Service (SUS) at risk. Another study that examined 4180 users of the SUS website from across the country demonstrated that Brazilians with access to online social networks have basic knowledge about COVID-19 and can identify false information. Respondents knew more about prevention, transmission, and social distancing, but had difficulty in identifying symptoms, risk groups, and correct conduct in case of infection. Men, the elderly and people with low education or from places with a lower human development index found it more difficult to detect false information.^26^

In correlated efforts to curb the effects of misinformation, a multicenter study was conducted to examine the impact of a digitally delivered intervention where medical students from 12 colleges demystified fake news and responded to COVID-19 questions from 375 elderly residents. The initiative highlighted how interpersonal communication facilitated by digital channels like WhatsApp could enable a greater understanding of the risks of misinformation among the elderly population.^26^ While these programs suggest disparate institutional efforts to curb online COVID-19 misinformation, we are interested in the dynamics of individual-level actions undertaken to tackle this problem.

## MATERIALS AND METHODS

### Study design

In this cross-sectional study, we analyzed a subset of questions enshrined within a larger dataset of a Portuguese-language online survey of WhatsApp users in Brazil,^8^ assessing their responses to COVID-19 misinformation and corrective information. As part of this online survey, a convenience sample of WhatsApp users from Brazil was recruited online by Qualtrics (a global professional survey firm headquartered in the United States) between May 26 and June 10, 2020. All participants were required to be adult WhatsApp users (18 years of age or over) who had heard of COVID-19. In the survey questionnaire, we also asked participants about their sex, age, highest level of education attained, household income, and time spent using WhatsApp to discuss COVID-19 (see Table 1 in Findings section for detailed participant profiles).

**Table 1.** Participant demographic profile and descriptive statistics of key independent variables of interest (*N *=* *726)^a^

Variables	Categories	*n*	%
Age	18–54	360	49.6
	55+	366	50.4
Sex	Male	428	59.0
	Female	298	41.0
Education	<Undergraduate degree	328	45.2
	≥Undergraduate degree	398	54.8
Monthly household income	R$2999 and less	261	36.0
	R$3000–6999	290	39.9
	R$7000 or more	175	24.1
Location	North	15	1.9
	North-east	173	23.9
	Mid-west	34	4.7
	South	104	14.0
	South-east	405	55.6
Misinformation exposure^b^	COVID-19 does not spread in hot weather	474	65.3
	Hot foods and drinks can protect you from COVID-19	300	41.3
	COVID-19 vaccines already exist	312	43.0
	Gargling saltwater/vinegar can protect you from COVID-19	332	45.7
	Hot pineapple can cure COVID-19	128	17.6
Time discussing COVID-19 on WhatsApp	No time spent	267	9.2
	<1 hour	267	36.8
	1–3 hours	257	35.4
	>3 hours	135	18.6

a Data for this table were sourced from Figure 2 in Vijaykumar et al.^8^ under http://creativecommons.org/licenses/by/4.0/.

b Frequencies denote the number of participants who responded “yes” when asked whether they had come across each of these statements.

**Table 2.** Summary statistics of scales used in the analyses

Variable	M	SD	Cronbach’s α
Misinformation belief	1.58	0.80	.83
Critical message evaluation	3.34	1.05	.86
WhatsApp information seeking	3.23	1.08	.82
Perceived severity	4.43	0.73	.90
Perceived susceptibility	3.48	0.92	.85
Correction to group	3.73	0.92	.81
Correction to sender	3.90	0.88	.66
Passive/no correction	2.22	0.84	.54

### Measures

#### Misinformation exposure and beliefs

Misinformation exposure and beliefs were assessed using 5 examples of misinformation messages that were circulating on social media in Brazil immediately prior to the start of our data collection. Examples of these messages included: “Coronavirus (COVID-19) does not spread in places with warm/hot weather,” “You can protect yourself from coronavirus if you eat hot food or drink hot water,” and “Hot pineapple water can cure coronavirus.” Participants indicated “Yes” or “No” as to whether they had seen these messages on WhatsApp prior to taking part in the study, with more “Yes” answers indicating greater exposure to the misinformation message. Participants were then asked to rate the accuracy of the 5 statements individually using a 5-point Likert-type scale ranging from 1= “Completely inaccurate” to 5= “Completely accurate.”

Given that the COVID-19 situation at the time of data collection was still rapidly evolving, we leveraged the local expertise of one of our coauthors, a health communication researcher, to identify specific examples of misinformation. They provided 5 examples of misinformation that were circulating in Brazilian WhatsApp networks. The team verified each statement against the scientific consensus at the time to ensure that all the statements were indeed misinformation.

#### Health beliefs

Perceived severity of and perceived susceptibility to COVID-19 were both measured using a 3-item 5-point Likert scale adapted from Witte et al,^27^ ranging from 1 = “Strongly disagree” to 5 = “Strongly agree.” Specifically, perceived severity was measured with statements like “I believe Coronavirus (COVID-19) has serious negative consequences” (α = .69). Perceived susceptibility was measured with statements like “It is likely that I will get Coronavirus (COVID-19)” (α = .86).

#### COVID-19 information seeking on WhatsApp

A self-structured 3-item measure of information seeking on WhatsApp was created for this study (α = .82) and presented to participants to respond using a 5-point Likert scale ranging from 1 = “Strongly disagree” to 5 = “Strongly agree.” Items included statements like “I intend to seek Coronavirus (COVID-19) related information on WhatsApp frequently.”

#### Critical message evaluation

Critical message evaluation was measured using 5 items adapted from Scull et al^28^ on a 5-point Likert-type scale ranging from 1 = “Never” to 5 = “Always” (α = .86). Items included statements like “When I view social media messages posted by my friends, peers, or people like me, I think about the purpose behind the message/post.”

#### Time spent discussing COVID-19

Time spent discussing COVID-19 was measured using a single item “How much time do you spend looking at or discussing COVID-19 information on WhatsApp each day?,” on a 5-point Likert-type scale where 1 = “no time spent at all,” 2 = “less than one hour,” 3 = “between one and three hours,” 4 = “between three and five hours,” and 5 = “more than five hours.” Points 4 and 5 were combined for analysis to maintain similar proportions of the sample in each of the categories (see Table 3 for frequencies).

**Table 3.** Principal component analysis identifying 3 distinct types of social correction behaviors

Social correction behaviors	Factor loading			Summary statistics	
	1	2	3	α	*M* (SD)
Factor 1: Active correction to group				.81	3.73 (0.92)
Inform the whole group that the forward had inaccurate information	**0.75**	0.13	−0.29		
Address the sender individually but send the message to the entire group	**0.84**	0.04	0.13		
Supply the accurate information to the whole group	**0.71**	0.21	−0.33		
Address the sender individually but supply the accurate information to the entire group	**0.83**	0.09	0.15		
Factor 2: Active correction to sender				.66	3.90 (0.88)
Inform the sender immediately	0.26	**0.54**	−0.42		
Inform the sender privately/separately that the forward had inaccurate information	−0.03	**0.84**	0.03		
Supply the accurate information to the sender privately/separately	0.20	**0.76**	−0.11		
Factor 3: Passive/no correction				.54	2.22 (0.84)
Inform the sender after waiting for a while	0.25	0.32	**0.51**		
Not inform the sender at all	−0.08	−0.12	**0.82**		
Take no action at all	−0.12	−0.18	**0.77**		

*Notes:* This table displays the findings of the PCA conducted on the 10 items used to assess feedback response to forwarded COVID-19 messages. The table shows 3 key factors which were identified following this analysis and the relevant reliability and descriptive statistics for these factors. Values in bold indicate the best fit for each item on to the 3 factors.

PCA: principal component analysis.

#### Social correction behaviors

Based on the conceptual framework, a self-structured 10-item 5-point Likert scale (1 = “Strongly disagree” to 5 = “Strongly agree”) comprising different combinations of whether or not participants would send a correction, when they would send the correction and to whom they would send it were developed. For instance, one of the question stems said: “If you find that there is incorrect or fake COVID-19 misinformation in a WhatsApp forward you have just received you will…,” with the following responses: inform the sender immediately/inform the sender after waiting a while/not inform the sender at all. A complete list of statements is available in Table 2.

### Procedure

Ethical approval for the study was granted by a research university in the United Kingdom. Participants were recruited online by Qualtrics through multiple platforms: social media, e-mail invitations to propriety panels, and online advertising (including online survey platforms). An anonymous link to the study survey was used in all recruitment methods. Participants received remuneration for their participation based on how they were recruited, some received points that could be redeemed for items whilst others were directly reimbursed the monetary value for participation. Participants were shown an information sheet outlining the nature of the study and informing them of what they would be required to do, if they consented. Those who did not consent to take part in the research were skipped to the end of the survey. Those who consented first provided demographic information. Participants then completed the survey in the order described in the Questionnaire section before being debriefed. Contact details for the principal researcher were included in the information and debrief sheet should participants need further information, prior, during, or after their participation in the study. Participants took approximately 10–15 minutes to complete the survey.

### Data analyses

All data were analyzed using SPSS version 27.^29^ Descriptive statistics were first calculated for the predictor and control variables in the study (see Table 1). Three participants who identified their sex as “other” were excluded from the analysis. The consideration of gender minority communities was outside the scope of this study but merits future investigation. The final analysis thus accounted for only male and female respondents, with gender being treated as a continuous variable.^30^

We performed a principal component analysis (PCA) using orthogonal rotation on the 10 SCB items (see Table 3 for results and new factors). Independent samples *t* tests were then used to determine any statistical differences between any of the control predictors with 2 levels (see Table 4) and a one-way independent groups Analysis of Variance (ANOVA) for control predictors with 3 levels (see Table 5) and the dependent variables. Predictor variables were then input into a correlation matrix (see Supplementary Information) to assess any multicollinearity issues. Finally, 3 hierarchical regressions (see Table 6) were run (one for each of our dependent variables) to ascertain which of the variables significantly contributed to the final models.

**Table 4.** *T* test findings highlighting differences between age, sex, and education across dependent variables: active group or private feedback and passive/no feedback

Social corrective behaviors	Predictors	Age		*T* test			Sex		*T* test			Education		*T* test		
	Levels	18–54 (*N *=* *360)	55+ (*N *=* *366)	*t*	*P*	*d*	Males (*N *=* *428)	Females (*N *=* *298)	*t*	*P*	*d*	<UG (*N *=* *328)	≥UG (*N *=* *398)	*t*	*P*	*d*
Active group feedback		3.71 (0.96)	3.71 (0.87)	−0.65	.51	.04	3.79 (0.89)	3.65 (0.94)	2.02	.04*	.15	3.68 (0.89)	3.77 (0.93)	−1.35	.18	.10
Active private feedback		3.90 (0.93)	3.90 (0.83)	−0.10	.92	<.01	3.88 (0.88)	3.93 (0.88)	−0.72	.47	.06	3.82 (0.89)	3.97 (0.87)	−2.40	.02*	.17
Passive/no feedback		2.33 (0.88)	2.12 (0.78)	3.47	<.01**	.25	2.26 (0.84)	2.17 (0.84)	1.45	.15	.11	2.28 (0.82)	2.18 (0.85)	1.60	.11	.12

*Notes:* In the *T* test statistic columns, *t* refers to the t statistic, *P* refers to the significance of the tested difference <.05 denotes a significant difference (<.05* and <.01**), *d* refers to the Cohen’s *d* a way of measuring the size of the effect found.

**Table 5.** ANOVA findings highlighting differences between monthly income brackets and the dependent variables: active group or private feedback and passive/no feedback

Social corrective behaviors	Monthly household income			ANOVA statistics			
	≤R$2999 (*N *=* *261)	R$3000–R$6999 (*N *=* *290)	≥ R$7000 (*N *=* *175)	*df*	*F*	*P*	*η^2^s*
Active group feedback	3.68 (0.88)	3.81 (0.93)	3.67 (0.95)	2, 723	1.76	.17	0.01
Active private feedback	3.83 (0.92)	3.95 (0.86)	3.90 (0.88)	2, 723	1.48	.23	<0.01
Passive/no feedback	2.26 (0.83)	2.23 (0.88)	2.16 (0.84)	2, 723	.74	.48	<0.01

*Notes:* In the ANOVA statistic columns, *df* refers to the degrees of freedom, *F* refers to the F statistic, *P* refers to significance of the tested difference <.05 denotes a significant difference (<.05* and <.01**), *η^2^s* (*partial eta squared)* is a measure of effect size for the independent groups ANOVA.

**Table 6.** Regression models showing standardized beta-weights for factors that predict social correction behaviors

Block	Variable	Correction to group	Correction to sender	Passive or no correction
1 (Demographic)	Age (55+)	.03	−.01	−.06
	Sex	−.08*	.03	−.01
	Education (UG+)	.03	.05	−.01
	Household income	−.06	−.01	.00
2 (Misinformation)	Misinformation exposure	.04	.02	−.00
	Misinformation belief	.01	.03	.11**
3 (Technological)	Information seeking on WhatsApp	.20**	.21**	.14**
	Critical message evaluation	.15**	.10**	−.13**
	Time discussing COVID-19	−.01	.11**	.14**
4 (Health beliefs)	Perceived severity	.07	.14**	−.20**
	Perceived susceptibility	.03	−.05	.02
				
5 (Correction behaviors)	Correction to group	−	.20**	−.00
	Correction to sender	.22**	–	−.17**
	Passive or no correction	−.00	−.15**	−
	*R* ^2^	.19**	.24**	.17**
	*N*	726	726	726
				

* *P* < .05,

** *P* < .01.

## RESULTS

### Recruitment and response

We initially recruited 1100 participants of whom 197 did not provide consent, 102 were not WhatsApp users, and 162 were excluded as duplicates, incomplete datasets, or for not completing the study within time parameters. Three participants who identified their sex as “other” were excluded from the analysis due to marginal representation. The final analysis accounted for *N* = 726 participants or 66% of the initially recruited sample.

### Participant profile 

Our sample was predominantly male with a majority of the participants having completed at least an undergraduate degree (55%) and most (∼40%) belonging to the middle-income bracket (R$3000–6999) (Table 1). Exposure to misinformation about COVID-19 not spreading in hot weather was the highest (65.3%) and the curative powers of pineapple were the lowest (17.6%). More than 7 in 10 participants spent fewer than 3 hours discussing COVID-19 on WhatsApp every day. The perceived severity of COVID-19 among the participants was higher than perceived susceptibility to being infected by it.

### Scale statistics

Means, standard deviations, and reliability scores for all scales are presented in Table 2. Overall, we found low levels of misinformation belief among participants but high levels of perceived severity. All the scales used in the survey had high levels of reliability measured by Cronbach’s α ranging from .82 to .90.

### Social correction behaviors (RQ1)

The 10 items in the PCA loaded on to 3 distinct types of SCBs: (1) *Correction to Group*, which involved different versions of providing feedback to the whole group (eg, a group chat/message), (2) *Correction to Sender*, which involved providing feedback on the misinformation separately or individually to the sender only, and (3) *Passive/No Correction*, which involved not engaging in either of the above 2 SCBs (Table 3).

### Demographic differences (RQ2)

Our analyses revealed 3 significant differences (Tables 4 and 5). A significant effect of age on passive or no correction was found (*P* = .01): younger participants (18–54 years) (*M* = 2.33, SD = 0.88) were more likely to engage in passive or no correction than older participants (55+ years; *M* = 2.12, SD = 0.78). Education was found to have a significant effect on correction to the sender (*P* = .02); participants with an undergraduate level degree or higher (*M* = 3.97, SD = 0.87) were more likely to engage in correction to sender than those without an undergraduate degree (*M* = 3.82, SD = 0.89).

Finally, a statistically significant effect of sex was also found on correction to group (*P* = .04): male participants (*M* = 3.79, SD = 0.89) indicated a higher preference than female participants (*M* = 3.64, SD = 0.94) for engaging in correction to group. No significant difference of income was found for any of the dependent variables.

### Factors influencing SCBs (RQ3)

We performed hierarchical linear regression analysis to separately analyze predictors of the 3 SCBs while controlling for demographic variables (age, sex, education, and income; Table 6). The main predictors assessed were misinformation factors (exposure and beliefs), technological factors (information seeking on WhatsApp, critical message evaluation, and time spent discussing COVID-19 on WhatsApp), and health beliefs (perceived severity and perceived susceptibility) as the predictors (IVs). Initial correlation analysis (included in Supplementary Information) detected no issues with multicollinearity (*r* ≥ .80) between predictor variables, showing that the predictors were not strongly related to each other.

#### Correction to group

The final model was able to significantly account for 19% of the variance in correction to group (*R*^2^ = .19, *P* < .01). Of the predictors included in this model, information seeking on WhatsApp (*b* = .20, *P* < .01), critical message evaluation (*b* = .15, *P* < .01), and correction to sender (*b* = .22, *P* < .01) were found to be 3 positive predictors of correction to group: participants seeking more information on WhatsApp, conducting more critical message evaluation and being more likely to send corrective information privately to the sender, were more likely to engage in correction to group as a way of social correction. Sex was also found to be a significant predictor of correction to group: female participants reported a lesser preference to engage in group feedback than males (*b* = −.08, *P* = .03). Among the significant predictors, the standardized beta-weights suggest that correction to sender had the strongest relationship with group feedback, thus functioning as the strongest predictor for participants’ behavior surrounding correction to group.

#### Correction to sender

The final model was able to significantly account for 24% of the variance in correction to sender (*R*^2^ = .24, *P* < .01). Despite education having a significant effect on correction to sender prior to the regression (see “Results” for RQ2), education was not found to be a significant predictor on this SCB (*b* = .05, *P* =.15). Five of the remaining variables were found to be positive predictors of correction to sender: perceived severity (*b* = .14, *P* < .01), information seeking on WhatsApp (*b* = .21, *P* < .01), critical message evaluation (*b* = .10, *P* < .01), time spent discussing COVID-19 (*b* = .11, *P* = .01), and correction to group (*b* = .20, *P* < .01): participants perceiving higher COVID-19 severity, seeking more information on WhatsApp, conducting more critical message evaluation, spending more time discussing COVID-19, and more likely to send corrective information to a group, were more likely to engage in correction to sender. Passive or no correction was found to be a negative predictor of correction to sender (*b* = .15, *P* < .01). Information seeking on WhatsApp was found to be the strongest predictor of correction to sender.

#### Passive or no correction

The final model was able to significantly account for 17% of the variance in passive or no correction (*R*^2^ = .17, *P* < .01). Despite leading to significant differences in passive or no correction prior to the regression (see “Results” for RQ2), age was not found to be a significant predictor in this model (*b* = −.06, *P* =.10). Six significant predictors were found in this model. Three of these were positive predictors: misinformation belief (*b* = .11, *P* < .01), information seeking on WhatsApp (*b* = .14, *P* < .01), and time spent discussing COVID-19 (*b* = .14, *P* < .01) participants with more misinformation belief, seeking more information on WhatsApp, and spending more time discussing COVID-19 were more likely to engage in passive/no correction behavior. The other 3, critical message evaluation (*b* = −.13, *P* < .01), perceived severity (*b* = −.20, *P* < .01), and correction to sender (*b* = .17, *P* < .01) were found to be negative predictors: participants perceiving lower COVID-19 severity, conducting less critical message evaluation and corrections to sender were more likely to engage in passive/no feedback correction behavior. Standardized beta-weights suggest perceived severity was the strongest predictor in the model.

## DISCUSSION

We sought to understand how individual social media users might engage in SCBs pertaining to online misinformation and which demographic, health belief, and technological factors influence this behavior. Studies of peer-norms on social media in times of crises (including public health crises) reveal how networked peers might influence communication and various behavioral outcomes.^31–33^ We identified 3 distinct types of individual-level SCB: Correction to Group (sent to one’s WhatsApp group), Correction to Sender (correction sent only to the original sender of the misinformation), and Passive/No Correction. Our survey first revealed the pattern of how different demographics influenced the 3 types of SCB: first, younger participants exhibited greater passivity in engaging with social correction; second, higher educational attainment was associated with providing correction to the original sender; and third, male participants were more likely to send the correction to the entire group.

Information seeking and critical message evaluation significantly affected all 3 SCB types. Information seeking was positively associated with all 3 SCB types (ie, the more individuals seek information about COVID-19 on WhatsApp, the more likely they are to engage in both active and passive SCB). Given the nascence of this research area, our study does not offer immediate explanations for this finding. We speculate that this association might be related to the content of the COVID-19 information sought on WhatsApp or subsequent information processing, neither of which our study has not captured. Contrastingly, critical message evaluation is an important determinant of whether one engages in active (ie, group and sender corrections) or passive SCB (ie, no correction). Individuals who critically evaluate messages are thus more likely to correct misinformation via group- and/or sender-corrections on WhatsApp.

We also found that each SCB has a distinct strongest predictor: namely, correction to sender for correction to group (positive association), information seeking for correction to sender (positive association), and perceived severity for passive/no correction (negative association). These findings suggest that encouraging users to actively seek (accurate) health information could make them more likely to send corrections to the sender which emerged as the “anchor” SCB—meaning, it connected and helped predict other SCB types in positive (Correction to Group) and negative (Passive/No Correction) directions, respectively. It seems that misinformation correction among Brazilian WhatsApp users is a cultivated process: those who are keen on correcting the sender alone might be encouraged or facilitated to share their corrections more publicly, in a group setting. In terms of passive SCB, we find that an elevated perception of the severity of the health risk might discourage users from engaging in SCBs. From a health risk communication standpoint, this finding highlights the importance of calibrating messages around the severity of health risks in a manner that is commensurate with the actual level of risk.

Interestingly, while the predictive power of age and education seemed to be diminished in the hierarchical regression model when entered as control variables, sex remained a stand-alone significant predictor of SCB (ie, female participants were more reluctant to engage in providing social correction to the group). If we consider the role of normative beliefs in such contexts, it is possible that the prospect of being negatively perceived by the group discouraged female participants from engaging in SCB to the group. This sex-based behavioral pattern in group correction merits further theoretical investigation as well as practical considerations about motivating female social media and mobile app users to engage in more public social correction with higher visibility and group impact.

As a relatively understudied strategy for correction of misinformation, social correction can be positioned between self-correction via information-vetting^34^ and external correction routes via government health agencies and news media. Social correction can be an effective supplemental correction strategy to help amplify public health authorities’ misinformation debunking efforts and news media’s fact-checking measures. Depending on the features and characteristics of different social media platforms, whether social correction is provided to the misinformation sender privately or in a group has implications on the process and magnitude of impact of such correction efforts. Such efforts can be properly enabled or facilitated by social media platforms to unlock the persuasive power of social media peers when they exert their influence based on factual information and the motivation to correct people in their networks.^13^

In terms of health beliefs, we found that perceived severity was a powerful predictor of all 3 feedback routes of social correction among WhatsApp users. Campaigns to help enhance the perception of threat severity could help with social correction in small networks like WhatsApp. We found that critical message evaluation was important amongst the technological factors. Message evaluation, which is important in the primary information vetting process,^34^ seemed to lead the user toward providing feedback. Because of this, our findings highlight the need to build critical thinking and informational literacy skills among social media users.

Spending a greater amount of time on WhatsApp discussing COVID-19 was significantly associated with providing correction to the sender, and negatively associated with providing no correction. Therefore, it appears that the time spent deliberating over and discussing information received on WhatsApp facilitates peer-to-peer dialog-based social correction. The private route, although not amplifying the correction in a group setting, might encourage the sender to consider vetting the information further (even revising their own original judgment on the information and themselves), thus initiating secondary information vetting (*ibid*).

Misinformation belief was significantly associated with “passive/no correction” but not with “active correction.” This finding implies that those with high belief in misinformation are less inclined to engage in SCB while those with low belief in misinformation are more inclined to do so. Such a scenario would be useful in minimizing situations where scientifically accurate information might be erroneously corrected by group members with high belief in misinformation. This finding strengthens the case for interventions that can bolster SCB by building skills to differentiate between accurate information and misinformation.

This suggests that awareness about one’s beliefs in misinformation might prove to be a deterrent against practising active SCBs. It is possible that deeply entrenched misinformation beliefs might undermine such an inference and is beyond the scope of the study. However, if our finding is supported by future research, such a deterrent would be especially useful in preventing instances where social media users might end up correcting content that might, in fact, be based on scientific consensus. In such a context, our findings strengthen the case for interventions that strengthen the ability of social media users to recognize misinformation and their belief in it which, in turn, might shape healthier SCBs.

Lastly, we found moderate to high levels of SCB to the group and privately to the sender. This finding indicates an important opportunity for Brazil’s public health establishment to leverage in terms of combating COVID-19 misinformation. Our inference from this finding is consistent with the assertion that Brazil’s social media users might benefit from resources by public health agencies that provide them with evidence that are attributed to specific sources.^24^^,^^25^ These resources could further strengthen social media users’ ability to function as community-based misinformation watchers and alleviate the burden on fact-checking agencies. Equally, these fact-checking agencies could help develop community capacity in identifying misinformation, using online information verification tools, and generating source-based corrections that could be sent by users.^35^

Our study has several limitations that constrain the generalizability of our findings and could be addressed by future research. First, the current study only looked at 2 elements of the health belief model (HBM), perceived severity and perceived susceptibility. Other HBM elements, such as perceived benefits and barriers of preventive action and perceived self-efficacy, need to be further examined in future studies applying the full HBM model to studies of managing the “infodemic” (spread of excessive and false information).^2^ Second, this study was conducted at a single point in time. To enhance ecological validity, future studies should consider using a longitudinal design to compare the pre- and-posteffects of each point of corrective communication and multiple exposures to misinformation and corrective information. How the state of COVID-19 (mis)information evolves, exerting different impacts on behavioral outcomes at different phases or stages of the pandemic, needs to be further examined by longitudinal studies, especially how individuals respond to and manage informational uncertainty and complexity in the context of a dynamic public health crisis.^36^ Third, our study sample is not representative of the Brazilian population. The generalizability of the findings in future research studies may be enhanced using representative samples with stratified sampling strategies. Fourth, misinformation exposure is limited to a potentially arbitrary selection of misinformation messages found on social media. As the quality and nature of the content of each message differ, subsequent SCB may vary depending on the quality of content to which users are exposed. The frequency of exposure to such messages was not captured in the current study. In addition, temporal factors (eg, the time from exposure to content on social media and later peer interaction on social networking platforms like WhatsApp) that may influence peer interaction behaviors need to be investigated by future studies. Lastly, the definition of misinformation we employed—as any claim that is false based on current scientific consensus—must be consumed with caution for 2 reasons. One, it may be challenging to measure consensus among scientists at any one point in time. And two, if deployed loosely and prematurely, the consensus can be exclusionary of other less well-held perspectives that may be more evidentiarily robust and, in doing so, create new ground for misinformation.

## CONCLUSION

More than a year after the COVID-19 pandemic, online misinformation continues to impede the public response to vaccination programs by exacerbating vaccine hesitancy and denial through conspiracy theories and misleading claims about safety and side-effects.^37–39^ In this scenario, when public health systems are stretched, sometimes to the limit, individual-level behaviors to combat misinformation could complement systems-level infodemic management efforts. Our study adds new elements to these efforts by identifying factors related to health belief and social media use, along with key demographic factors and misinformation belief, as essential drivers that explain and predict infodemic management outcomes at the levels of the individual user and their social media community. Our findings also highlight the importance of activating all viable routes for misinformation correction, unlocking the power of social correction as conduit linking the influence of self-correction via information vetting and corrective communication and fact-checking efforts initiated by public health authorities and news media. We also note the responsibility of public and private health institutions to invest in information literacy initiatives by distilling complex scientific jargon into more accessible content and infuse a greater sense of personal responsibility in being vigilant to COVID-19 misinformation circulating on WhatsApp and other social networks.

## FUNDING

This work was supported by Facebook Inc. through the WhatsApp Social Science Misinformation Research Award.

## AUTHOR CONTRIBUTIONS

SV led the conceptualization and writing. DTR led the methodology, statistical analysis, and reporting of findings. YJ contributed to conceptualization and writing. MSdOC contributed to writing the manuscript.

## SUPPLEMENTARY MATERIAL

Supplementary material is available at *Journal of the American Medical Informatics Association* online.

## Supplementary Material

ocab219_Supplementary_Data
