# Supplementary material for: Dynamics of social corrections to peers sharing COVID-19 misinformation on WhatsApp in Brazil
Source: J Am Med Inform Assoc. 2021 Nov 22;29(1):33–42. doi: 10.1093/jamia/ocab219 (PMC8586730; doi:10.1093/jamia/ocab219)
Supplement: ocab219_Supplementary_Data [file ocab219_supplementary_data.zip › ocab219-suppl_data/Supplementary Information Revised.docx]

**Supplementary Information**

*(This appendix has been back-translated from the Portuguese version of the survey that was originally administered to all survey respondents)*

| **Questions** | **Answer**  **Options** | **Chronbach’s α** |
| --- | --- | --- |
| **(Misinformation Exposure) \|**  **Please indicate if you have seen this type of message on WhatsApp.** | | |
| Coronavirus does not spread in places with warm/hot weather | Yes/No | NA |
| You can protect yourself from coronavirus if you eat hot food or drink hot water |  |  |
| A vaccine for Coronavirus is already available |  |  |
| Salt-water and/or vinegar gargling can protect you from coronavirus |  |  |
| Hot pineapple water can cure coronavirus |  |  |
| **(Misinformation Beliefs) \|**  **Please rate the accuracy of the following statements** | | |
| Coronavirus does not spread in places with warm/hot weather | 1 = Completely inaccurate to  5 = Completely accurate | 0.83 |
| You can protect yourself from coronavirus if you eat hot food or drink hot water |  |  |
| A vaccine for Coronavirus is already available |  |  |
| Salt-water and/or vinegar gargling can protect you from coronavirus |  |  |
| Hot pineapple water can cure coronavirus |  |  |
| **(Perceived Severity) \|**  **Please indicate how much you agree with the following statements.** | | |
| I believe Coronavirus (Covid-19) is severe. | 1 = Strongly Disagree to  5 = Strongly Agree | 0.90 |
| I believe Coronavirus (Covid-19) has serious negative consequences. |  |  |
| I believe Coronavirus (Covid-19) is not serious as media says. |  |  |
| **(Perceived Susceptibility) \|**  **Please indicate how much you agree with the following statements.** | | |
| It is likely that I will get Coronavirus (Covid-19). | 1 = Strongly Disagree to  5 = Strongly Agree | 0.85 |
| I am at risk of getting Coronavirus (Covid-19). |  |  |
| It is possible that I will get Coronavirus (Covid-19). |  |  |
| **(COVID-19 Information Seeking on WhatsApp) \|**  **Please indicate how much you agree with the following statements.** | | |
| I intend to seek Coronavirus (COVID-19) related information on WhatsApp frequently. | 1 = Strongly Disagree to  5 = Strongly Agree | 0.82 |
| I will consider others' Coronavirus (COVID-19) experience on WhatsApp before I make decisions regarding COVID-19. |  |  |
| I will ask others on WhatsApp to provide me with their suggestions before I make decisions regarding Coronavirus (COVID-19). |  |  |
| **(Critical Message Evaluation) \|**  **Please rate the following statements on a scale from 'Never' to 'Always'.**  **When I see messages on social networks posted by my friends, colleagues or people like me, I think...** | | |
| Critical Message Evaluation | 1 = Strongly Disagree to 5 = Strongly Agree | 0.86 |
| The purpose behind a message/post. |  |  |
| Who created a message I see on social media. |  |  |
| What people who made a media message want me to believe. |  |  |
| The things that advertisers do to get my attention. |  |  |
| Whether the things that message senders want me to do are good for me. |  |  |
| **Your Age** | - 18-24 years - 25-34 years - 35-44 years - 45-54 years - 55-64 years - 65-75 years - 75+ years | NA |
| **What state do you currently live in?** | - AC - AL - AM - AP - BA - CE - DF - ES - GO - MA - MG - MS - MT - PA - PB - PE - PI - PR - RJ - RN - RO - RR - RS - SC - SE - SP - TO | NA |
| **Please select only one option for each question.**  **Your gender** | - Male - Female - Other | NA |
| **What is the highest level of education you have completed?** | - Elementary School - High school - Technical course - University Graduate - Master’s degree - Doctorate Degree | NA |
| **Your approximate monthly household income** | - R$1,000-R$2,999 - R$3,000-R$4,999 - R$5,000-R$6,999 - R$7,000 or more | NA |

**Social Correction Behaviors (Skewness)**

| **Factor 1: Correction to Group** (α=.81) | **Skewness** |
| --- | --- |
| *Inform the whole group that the forward had inaccurate information* | -.96 |
| *Address the sender individually but send the message to the entire group* | -.37 |
| *Supply the accurate information to the whole group* | -1.19 |
| *Address the sender individually but supply the accurate information to the entire group* | -.43 |
| **Factor 2: Correction to Sender** (α=.66) |  |
| *Inform the sender immediately* | -1.26 |
| *Inform the sender privately/separately that the forward had inaccurate information* | -.87 |
| *Supply the accurate information to the sender privately/separately* | -.87 |
| **Factor 3: Passive/No Correction** (α=.54) |  |
| *(2) Inform the sender after waiting for a while* | -.03 |
| *(3) Not inform the sender at all* | 1.13 |
| *(10) Take no action at all* | 1.35 |

**Correlation matrix for Principal Component Analysis**

|  | 1 | 2 | 3 | 4 | 5 | 6 | 7 | 8 | 9 | 10 |
| --- | --- | --- | --- | --- | --- | --- | --- | --- | --- | --- |
| Item 1 | - | .07 | -.36** | .33** | .40** | .18** | .36** | .38** | .16** | -.32** |
| Item 2 |  | - | .20** | .15** | .10** | .19** | .07 | .09* | .20** | .14** |
| Item 3 |  |  | - | -.07 | -.25** | -.01 | -.18** | -.28** | -.00 | .55** |
| Item 4 |  |  |  | - | .12** | .07 | .50** | .15** | .09* | -.14** |
| Item 5 |  |  |  |  | - | .51** | .24** | .63** | .43** | -.26** |
| Item 6 |  |  |  |  |  | - | .20** | .41** | .68** | -.05 |
| Item 7 |  |  |  |  |  |  | - | .33** | .27** | -.21** |
| Item 8 |  |  |  |  |  |  |  | - | .48** | -.32** |
| Item 9 |  |  |  |  |  |  |  |  | - | -.03  - |
| Item 10 |  |  |  |  |  |  |  |  |  |  |

| **. Correlation is significant at the 0.01 level (2-tailed). |
| --- |
| *. Correlation is significant at the 0.05 level (2-tailed). |

**Correlation Matrix for Regression Analysis**

|  | Predictors | | | | | | | | | | |  |  |  |
| --- | --- | --- | --- | --- | --- | --- | --- | --- | --- | --- | --- | --- | --- | --- |
|  | (1) | 2 | 3 | 4 | 5 | 6 | 7 | 8 | 9 | 10 | 11 | 12 | 13 | 14 |
| Age (1) | - | .03 | .04 | .11 ** | .06 | -.07 * | -.10 ** | -.10 ** | -.08 * | .12 ** | -.13 ** | .02 | .00 | -.13 ** |
| Sex (2) |  | - | .01 | -.07 * | .10 ** | -.08 * | .08 * | -.08 * | -.09 ** | .02 | .06 | -.08 * | .03 | -.05 |
| Education (3) |  |  | - | .43 ** | .03 | .14 ** | .01 ** | -.19 ** | -.04 | .20 ** | .14 ** | .05 | .09 * | -.06 |
| Income (4) |  |  |  | - | .05 | .13 ** | .06 | -.17 ** | .03 | .17 ** | .10 ** | .01 | .05 | -.04 |
| P. Severity (5) |  |  |  |  | - | .18 ** | -.02 | -.27 ** | .07 * | .16 ** | .08 * | .15 ** | .23 ** | -.26 ** |
| P. Susceptibility (6) |  |  |  |  |  | - | .14 ** | -.10 ** | .07 * | .13 ** | .11 ** | .09 * | .04 | -.02 |
| Misinformation Exposure (7) |  |  |  |  |  |  | - | .01 ** | .04 | .11 ** | .17 ** | .07* | .07 | .03 |
| Misinformation Belief (8) |  |  |  |  |  |  |  | - | .17 ** | -.09 ** | -.02 | .01 | -.02 | .20 ** |
| Information Seeking on WhatsApp (9) |  |  |  |  |  |  |  |  | - | .09 ** | .28 ** | .29 ** | .30 ** | .13 ** |
| Critical Message Evaluation (10) |  |  |  |  |  |  |  |  |  | - | .15 ** | .23 ** | .23 ** | -.18  ** |
| Time Discussing COVID-19 (11) |  |  |  |  |  |  |  |  |  |  | - | .12 ** | .21 ** | .12 ** |
| Correction to Group (12) |  |  |  |  |  |  |  |  |  |  |  | - | .33 ** | -.05 |
| Correction to Sender (13) |  |  |  |  |  |  |  |  |  |  |  |  | - | -.17 ** |
| Passive/ No Correction (14) |  |  |  |  |  |  |  |  |  |  |  |  |  | - |

(*p<.05, **p<.01)

**Handling Duplicates**

Duplicates are flagged by Qualtrics using their RelevantID software. According to Qualtrics, Relevant ID improves fraud detection by assessing respondent metadata to determine the likelihood that the same respondent is answering over and over; Relevant ID does not necessarily check the content of the responses for duplicates, since respondent can answer multiple times while giving different answers. This feature has three fields associated with it that you can report on: Q_RelevantIDDuplicate, Q_RelevantIDDuplicateScore, and Q_RelevantIDFraudScore. These fields are calculated using RelevantID technology. This technology checks if the respondent is cheating by taking the survey multiple times or whether a survey taker is fraudulent by analyzing a user’s browser, operating system, and location to provide a fraud score. Respondents are prevented from accessing the link multiple times by default using survey options, however there are ways that respondents can get around that such as incognito mode, clearing browser cookies, using VPNs. RelevantID is a more advanced way of flagging duplicates.
