# Supplementary material for: Dynamics of social corrections to peers sharing COVID-19 misinformation on WhatsApp in Brazil
Source: J Am Med Inform Assoc. 2021 Nov 22;29(1):33–42. doi: 10.1093/jamia/ocab219 (PMC8586730; doi:10.1093/jamia/ocab219)
Supplement: ocab219_Supplementary_Data [file ocab219_supplementary_data.zip › ocab219-suppl_data/Title Page.docx]

**Dynamics of Feedback Behaviours to Social Peers Sharing COVID-19**

**Misinformation on WhatsApp in Brazil**

Author 1: Santosh Vijaykumar

NB 138, Northumbria University

Newcastle upon Tyne NE1 8ST, United Kingdom

Email: [santosh.vijaykumar@northumbria.ac.uk](mailto:santosh.vijaykumar@northumbria.ac.uk), Telephone: (+44) 7393570480

Author 2: Daniel Rogerson

COCO Lab, Northumbria University

Newcastle upon Tyne NE1 8ST, United Kingdom

Email: [daniel.t.rogerson@northumbria.ac.uk](mailto:daniel.t.rogerson@northumbria.ac.uk)

Author 3: Yan Jin

Journalism Building, Room 223-F

University of Georgia Grady

120 Hooper Street, Athens, Georgia, 30602-3018, USA

[yanjin@uga.edu](mailto:yanjin@uga.edu)

Author 4: Mariella Silva de Oliveira Costa

Avenida L3 Norte, s/n, Campus Universitário Darcy Ribeiro, Gleba A

CEP: 70.904-130 – Brasilia – DF, BRAZIL

Email: [mariella.costa@fiocruz.br](mailto:mariella.costa@fiocruz.br)

Keywords: misinformation; COVID-19; social media; correction; behavior; Brazil

Word Count: 3,348
